# The impact of periampullary diverticula on cannulation and adverse events in endoscopic retrograde cholangiopancreatography

**DOI:** 10.1177/17562848241279105

**Published:** 2024-10-05

**Authors:** Arvid Gustafsson, Bobby Tingstedt, Greger Olsson

**Affiliations:** Departments of Research and Development and Surgery, Central Hospital, Region Kronoberg, Strandvägen 8, Växjö SE-351 85, Sweden; Department of Clinical Sciences Lund, Surgery, Lund University and Skåne University Hospital, Lund, Sweden; Department of Clinical Sciences Lund, Surgery, Lund University and Skåne University Hospital, Lund, Sweden; Departments of Research and Development and Surgery, Central Hospital, Region Kronoberg, Växjö, Sweden

**Keywords:** adverse events, cannulation, duodenal diverticula, endoscopic retrograde cholangiopancreatography, ERCP, periampullary diverticulum

## Abstract

**Background:**

Periampullary diverticulum (PAD) is commonly encountered in endoscopic retrograde cholangiopancreatography (ERCP) procedures.

**Objectives:**

We sought to determine whether PADs are associated with a lower success rate of cannulation and an increased risk of adverse events.

**Design:**

A retrospective cohort study was conducted using prospectively gathered nationwide registry data.

**Methods:**

Using the Swedish registry for gallstone surgery and ERCP, we analyzed a cohort of 66,974 prospectively registered ERCP procedures performed in 2006–2021. The presence of PAD was divided into two groups based on the PAD type: Boix type 1 (the papilla located inside the PAD) and Boix types 2–3 (the papilla located either at the edge of the PAD or immediately adjacent to the PAD). The primary outcomes were the success rate of cannulation and overall adverse events within 30 days.

**Results:**

PADs were registered in 8130 (12.1%) of ERCPs included in the study population. In total, 2114 (3.9%) patients had Boix type 1 PAD, while 5035 (8.2%) patients had Boix type 2 or 3 PAD. The chance of successful cannulation was lower in patients with type 1 PAD compared to no PAD (80.1% vs 88.7%; odds ratio: 0.42, 95% confidence interval: 0.38–0.46). No differences were seen in overall adverse events or post-ERCP pancreatitis. Adverse events occurred in 14.6% of patients with PAD type 1 and 16.0% of patients with PAD type 2 or 3, compared to 16.5% of patients without a PAD.

**Conclusion:**

Cannulation appears less successful during ERCP when the papilla is located in the PAD (i.e., type 1). Adverse events seem not to increase with the presence of a PAD, but they could theoretically be influenced by the inability to cannulate.

## Plain language summary

How a pouch in the duodenum affects the difficulty and complications of an endoscopic procedure investigating the bile ducts

A duodenal diverticulum (pouch in the duodenum) is a common finding during endoscopic examinations. A diverticulum can make it harder to do an ERCP, which is an endoscopic procedure that examines the bile ducts. The diverticulum might make it harder to do an ERCP because it affects the opening where the bile is released into the intestine. If the bile duct opening is inside the diverticulum, it is classified as type 1. If it is on the edge or outside, it is classified as type 2-3. This study used Swedish registry data to see if these types of diverticula make ERCP harder and cause more problems after the procedure. 47,486 procedures were investigated, and 12.1% had a diverticulum. A type 1 diverticulum makes ERCP harder. There was no difference in complications between the types of diverticula and no diverticulum. This means a diverticulum does not raise the risk of complications during ERCP. However, for a type 1 diverticulum, not being able to perform ERCP may prevent complications.

## Introduction

A duodenal periampullary diverticulum (PAD) is defined as a protrusion of the mucosa and submucosa, typically found in the papillary region. Its prevalence ranges from 5% to 33% in patients undergoing endoscopic retrograde cholangiopancreatography (ERCP)^1,2^; it may be even more common in older patients,^3–5^ and it has been reported to be more prevalent in women.^6^ As PADs are associated with common bile duct (CBD) stones^5,7–10^ and cholangitis,^11^ it could be assumed to be more prevalent in patients who undergo ERCP than in the general population. In 2006, Boix classified PADs by whether the papilla is located inside the diverticulum (type 1), on its edge (type 2), or adjacent to it (type 3).^12^ The PAD itself is asymptomatic, but it may alter the anatomy of the duodenal region and hinder the identification and cannulation of the papilla.^13–16^

Whether PAD hinders cannulation or poses a risk of adverse events is a topic of ongoing debate.^1,2^ In a comparison of ERCP procedures for PADs across different time periods, evidence suggests that cannulation success rates were equalized after 2000,^2^ coinciding with the introduction of the sphincterotome for cannulation and other technical developments. As a result, most studies conducted after 2000 demonstrate no significant differences in the success of cannulation with or without a PAD.^4,5,7,17–19^ However, recent evidence comparing various types of PADs indicates an increased cannulation difficulty, especially for type 1 PADs.^13–16^ Moreover, it is unclear whether the presence of a duodenal diverticulum has an impact on adverse events during ERCP, with two meta-analyses presenting conflicting findings.^1,2^ These discrepancies suggest that endoscopists’ potential fears of complications when a diverticulum is present may be exaggerated.

Therefore, the objective of this study was to investigate the incidence of PAD in a national ERCP cohort. In addition, we sought to assess the impact of different subtypes of PADs on adverse events, as well as cannulation success rates during ERCP.

## Methods

### Study design and population

**Figure 1.**
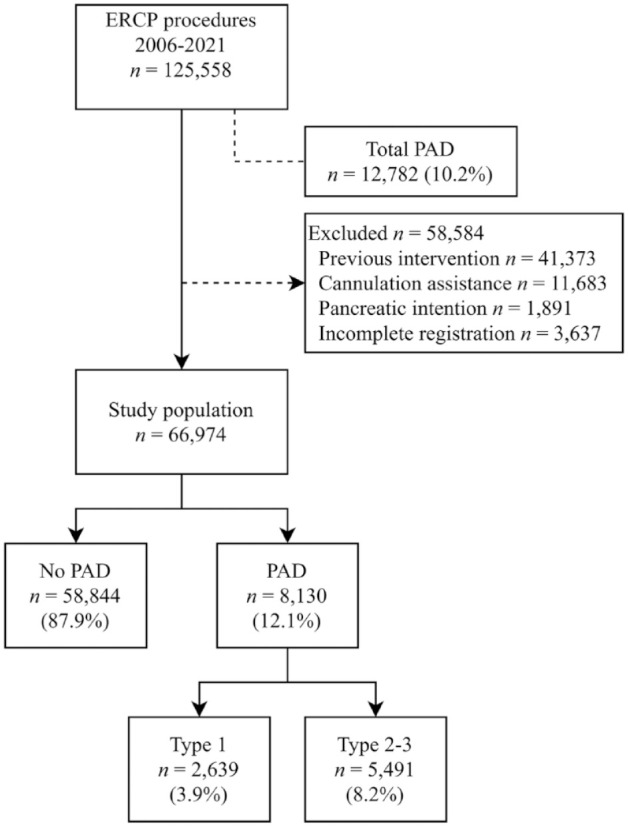
This flow chart illustrates the total number of procedures with PAD in the registry, the exclusion of certain procedures, and the categorization of PAD subtypes. PAD, periampullary diverticulum.

We conducted a nationwide population-based cohort study of all ERCP procedures included in the Swedish registry for gallstone surgery and ERCP (GallRiks) from 2006 to 2021. The procedures were consecutively performed. Patients who underwent ERCP and had a PAD were compared to those without a PAD in terms of cannulation success rate and 30-day overall adverse events. These outcomes were also compared across three groups: Boix type 1 PAD (the papilla located in the PAD), Boix types 2–3 PAD (the papilla located at the edge of or adjacent to the PAD), and no PAD.^12^ To ensure homogeneity among the patients undergoing ERCP procedures, only those with an intact papilla were included in the study. Accordingly, exclusion criteria comprised individuals who had undergone prior sphincterotomy, previously received stent placement, undergone rendezvous procedures, intention to cannulate the pancreatic duct, or received any other type of cannulation assistance (by percutaneous biliary drainage or endoscopic ultrasound). We also excluded procedures with incomplete registration and 30-day follow-up (Figure 1). In addition, we analyzed secondary outcomes regarding specific adverse events, such as perforation, pancreatitis, bleeding, and cholangitis. The study was appraised using the Strengthening the Reporting of Observational Studies in Epidemiology checklist for cohort studies.^20^

### The GallRiks registry

GallRiks, the Swedish Registry of Gallstone Surgery and ERCP, was created in 2005 by the Swedish Surgical Society. This online nationwide registry receives support from the Swedish Board of Health and Welfare. It uses an internet platform (ucr.uu.se/gallriks) to which 76 centers are currently connected, representing over 90% coverage of all ERCPs performed in Sweden—a consistent trend since 2011.^21^ The GallRiks registry is a large database that records approximately 9000 ERCP investigations annually, and it currently contains almost 150,000 ERCP procedures. The data from ERCP procedures are recorded by the endoscopist using over 100 variables—such as patient demographics, procedure specifics, indications, and any procedural complications—soon after the procedure. The registry protocol is closed after the procedure, and after 30 days, an independent local coordinator reviews the relevant medical records for any adverse events, which are recorded in the registry. The registry is regularly validated by independent observers at each participating center, which lends credibility to its data: GallRiks has been shown to have over 97% agreement with medical records.^22^

### Variables

For the logistic regression model analyzing primary and secondary outcomes, we selected independent variables that we deemed clinically significant for the relevant outcome. Below, we present the essential study variables (in italics), which are based on their definitions in the GallRiks registry.

The registration of the presence and type of diverticulum during ERCP in GallRiks is optional. If the question on registration was left unanswered, this was interpreted as an indication that no diverticulum was present during the ERCP procedure. The registry only allows for the recording of two types of PAD: Boix type 1, or Boix types 2–3. Therefore, it is not possible to differentiate between Boix type 2 and Boix type 3. In addition, it is not possible to record if there are multiple PADs, nor is it possible to record the size.

*Successful cannulation* is operationalized as the ability to achieve deep contact with the bile duct for investigation or intervention. However, GallRiks does not define a failure as solely caused by the PAD; any reason for cannulation failure is presented under the same outcome parameter, regardless of whether it was caused by the PAD or by another factor.

*Age* was organized (binned) into four categories, represented as quartiles, for use in the regression models.

*ASA (American Society of Anesthesiologists) classification* was divided into two groups: ASA 1–2 and ASA 3–4.

*Procedure time* was also categorized into quartiles.

*Pre-cut sphincterotomy* was defined as a sphincterotomy performed prior to any contact being made with any duct (biliary or pancreatic). In Sweden, this is typically performed with needle-knife sphincterotomy, but the definition could also include a pancreatic sphincterotomy.

*Procedural complications* are identified by the endoscopist during the ERCP procedure and include bleeding requiring intervention, perforation, or contrast extravasation.

*Adverse events* are documented within 30 days post-treatment and consist of a variable summing all adverse events. Furthermore, distinct specific adverse events are also recorded.

### Statistical analysis

Continuous variables are reported as means with standard deviations (SD), while categorical variables are presented as absolute frequencies with percentages. Student’s *t-*test and Pearson’s Chi-squared test were used for continuous and categorical data, respectively. The presence of missing data was measured in each group. A two-sided *p*-value less than 0.05 was considered statistically significant. Kruskal–Wallis and Mood’s median test were used for continuous variables in comparisons of types of PAD. Odds ratios (OR) and their 95% confidence intervals (CI) were calculated using multivariable logistic regression analysis to evaluate the relationship between the presence of PAD, its type, and related outcomes. Directed acyclic graphs were constructed to investigate potential causal relationships among the variables in the model and to prevent the adjustment of collider variables. Logistic regression model assumptions were evaluated, and any potential interfering correlations were assessed using both Spearman’s correlation and linear regression with an analysis of variance inflation factor. Potential outliers were assessed using Mahalanobis distance. Backward stepwise regression was used to eliminate variables with α > 0.1. Age and sex were pre-selected for inclusion in all models. IBM SPSS Statistics version 29.0.0 (241) (IBM Corp., Armonk, NY, USA) was employed for the statistical analysis.

## Results

A total of 125,558 ERCP procedures were recorded in the GallRiks registry in 2006–2021; of these procedures, 12,782 (10.2%) had a registered PAD. After 58,584 procedures deemed unsuitable for analysis were excluded, the study population consisted of 66,974 procedures (Figure 1); of these analyzed ERCP procedures, 8130 (12.1%) had a registered PAD. Within this subset, 2639 (3.9%) were classified as Boix type 1 (the papilla located in the PAD) and 5491 (8.2%) were classified as Boix type 2 or 3 (the papilla located at the edge of or adjacent to the PAD). In terms of baseline characteristics, older age, higher ASA score, a higher proportion of female patients, a higher prevalence of CBD stones, and a higher incidence of cholangitis were observed in patients undergoing procedures with a PAD (Table 1). The presence of missing data was evenly distributed and was not included in any analysis. In terms of the procedural characteristics (shown in Table 2), these characteristics did not indicate a major difference in procedure time between the groups, but precut sphincterotomy was performed less frequently in the PAD group, and sphincterotomy was performed more frequently in the PAD type 2–3 group.

**Table 1.** Baseline characteristics.

Total, *n* = 66,974	PAD (all), *n* = 8,130 (12.1%)	PAD Type 1, *n* = 2,639 (3.9%)	PAD Type 2–3, *n* = 5,491 (8.2%)	No PAD, *n* = 58,844 (87.9%)
Sex, *n* (%)				
Female	4,661 (57.3)	1,517 (54.3)	3,400 (58.7)	31,117 (52.9)
Missing		0	0	15 (0.0)
Age, mean (SD), years^a^	76.6 (11.8)	77.4 (11.1)	76.2 (12.0)	68.2 (16.7)
Missing		6 (0.2)	4 (0.1)	150 (0.3)
ASA, *n* (%)				
1–2	4,904 (60.3)	1,577 (59.8)	3,327 (60.6)	38,744 (65.8)
3–4	3,226 (39.7)	1,062 (40.2)	2,164 (39.4)	20,100 (34.2)
Missing		0	0	0
Indication, *n* (%)				
CBD stone	4,007 (49.3)	1,319 (50.0)	2,688 (49.0)	21,589 (36.7)
Malign./jaundice	1,778 (21.9)	530 (20.1)	1248 (22.7)	23,313 (39.6)
Cholangitis	1,402 (17.2)	482 (18.3)	920 (16.8)	4,699 (8.0)
Pancreatitis	372 (4.6)	109 (4.1)	263 (4.8)	2,921 (5.0)
Missing		0	0	0
Acute	6,018 (74.0)	1,938 (73.4)	4,080 (74.3)	42,323 (71.9)
Elective	2,112 (26.0)	701 (26.6)	1,411 (25.7)	16,521 (28.1)
Missing		0	0	0

a Kruskal–Wallis test (displayed) p = 0.07, Mood’s median test p = 0.07.

ASA, American Society of Anesthesiologists classification; CBD, common bile duct; PAD, periampullary diverticulum; SD, standard deviation.

**Table 2.** Procedural characteristics.

Total, *n* = 73,937	PAD, *n* = 8,589 (11.6%)	PAD Type 1, *n* = 2,796 (3.8%)	PAD Type 2–3, *n* = 5,793 (7.8%)	No PAD, *n* = 65,348 (88.4%)
Papilla manipulation, *n* (%)				
Sphincterotomy	6,360 (78.7)	1,857 (71.0)	4,503 (82.4)	43,050 (74.2%)
*p*	**<0.001**	NA	NA	Ref
*p*	–	**<0.001**	**<0.001**	Ref
Precut	761 (9.4)	250 (9.6)	511 (9.3)	7,089 (12.2)
*p*	**<0.001**	NA	NA	Ref
*p*	NA	**<0.001**	**<0.001**	Ref
Procedure time, mean (SD), minutes	37.2 (23.3)	37.4 (22.8)	37.0 (23.5)	37.3 (24.8)
*p*	0.72	NA	NA	Ref
*p*^a^	NA	**0.03** (Adj. 0.09)	0.31 (Adj. 0.92)	Ref
Procedural complication, *n* (%)	248 (3.1)	95 (3.6)	153 (2.8)	1,767 (3.0)
*p*	0.81	NA	NA	Ref
*p*	NA	0.08	0.37	Ref

Bold values indicate statistical significance at the *p* < 0.05 level.

a Kruskal–Wallis test (displayed) *p* = 0.07, Mood’s median test *p* = 0.07.

NA, not applicable; PAD, periampullary diverticulum; SD, standard deviation.

**Figure 2.**
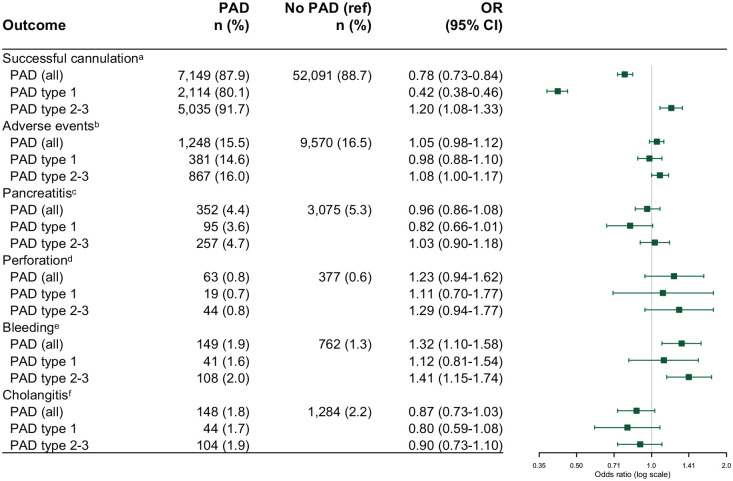
Odds ratio plot presenting the comparison of primary outcomes and specific adverse events between those with a PAD and those without. Furthermore, a separate comparison is displayed between various subtypes of PAD and no PAD. For details regarding the regression models see the Supplementary online material. ^a^Adjusted for sex, age, ASA, indication, acute, or elective setting. ^b^Adjusted for sex, age, ASA, precut, indication, acute, or elective setting, procedure time. ^c^Adjusted for sex, age, ASA, precut, indication, acute, or elective setting, procedure time. ^d^Adjusted for sex, age, precut, indication, procedure time. ^e^Adjusted for sex, age, ASA, precut, indication, acute, or elective setting, procedure time. ^f^Adjusted for sex, age, precut, indication, procedure time. CI, confidence interval; PAD, periampullary diverticulum.

In the regression model analyzing the effect of PAD and its subtypes on cannulation success, the final model found associated effects on successful cannulation for age, an acute setting, and the indications of CBD stone, cholangitis, and pancreatitis. The presence of a PAD showed a negative association with successful cannulation; however, this association was only observed in the group with a type 1 PAD, which had a cannulation rate of 80.1%. A type 2 PAD, however, showed a higher successful cannulation rate of 91.7% compared to both type 1 PAD and no PAD (Figure 2). The type 1 PAD failed cannulation rate represented an absolute risk reduction of 8.6% compared to no PAD (i.e., 8.6% fewer cannulated papillae of Vater).

Another regression model was constructed to analyze the effect of PAD on the outcome of overall adverse events. In the final model, neither PAD in general nor its subtypes showed differences from no PAD in overall adverse events (Figure 2). Associated effects on adverse events were observed for female sex, younger age, precut sphincterotomy, ASA 3–4, all indications versus jaundice, acute setting, and longer procedure time. For the secondary (i.e., specific) outcomes, separate regression models showed a higher rate (2%) of bleeding in type 2–3 PAD compared to the rate of 1.3% in no PAD. The specific separate outcomes are shown in Figure 2.

## Discussion

We identified an incidence of PAD of 10.2% in a nationwide population of over 125,000 ERCP procedures conducted in Sweden in 2006–2021, with approximately one-third of the papilla located in the PAD (Boix type 1). Previous reports have shown a wide range of PAD incidence, from 5% to 33%, with an 18% incidence in pooled data from a meta-analysis.^1,2^ Our reported incidence of 10.2% may be a more accurate representation of the true incidence of encountering a PAD during ERCP. Our study corroborates the known demographic findings that patients with PAD are typically older,^3–5^ more likely to be female,^6^ and have an increased risk of CBD stone and cholangitis.^5,7–11^

Our main findings indicate that the presence of PAD slightly decreases the chance of successful cannulation (87.9% vs 88.7%). This difference was primarily observed in cases with intradiverticular papilla (i.e., type 1), where only 80.1% could be cannulated; in contrast, for type 2–3 PAD cases, we found a higher rate (91.7%) of successful cannulation compared to ERCPs without PAD. These findings are somewhat inconsistent with a recent systematic review by Mu et al.,^2^ who found a higher incidence of cannulation failure in PAD but only in ERCP procedures performed before 2000. Their overall pooled cannulation rate for PAD from smaller studies was higher (95.4%) compared to our rate of 87.9%. However, in their subgroup analysis of studies reporting on intradiverticular papilla, they also found an association of this type of PAD with a higher incidence of cannulation failure, consistent with our findings. Another systematic review by Jayaraj et al.^1^ did not differentiate between different subtypes of PAD but reported a 50% reduced chance of successful cannulation for PAD in general, similar to our reported OR of 0.42 for type 1 PAD. The studies included in their review were conducted in and before the year 2000, which may reveal earlier difficulties in cannulating PAD in general, while advancements in ERCP cannulation techniques have made successful cannulation more likely—although our results suggest that this is not the case for type 1 PAD. This finding is consistent with more recent studies, which also show a pattern of unsuccessful cannulation for type 1 PAD.^14–16^ Our finding of a slight increase in successful cannulation in type 2 PAD was also shown by Yue et al.,^16^ but could also be explained by the control group possibly containing more difficult ERCPs. Regarding overall adverse events, we found no differences between patients with PAD—irrespective of type—and no PAD. These findings are consistent with the systematic review by Jajaray et al.^1^ and several recent studies.^5,7,14,15^ However, the systematic review by Mu et al.^2^ reported a higher risk of pancreatitis, bleeding, and perforation with the presence of PAD and other previous studies have reported mixed results, with an increased risk of post-ERCP pancreatitis, bleeding, and occasionally perforation; however, these studies were unable to differentiate between the different types of PAD in terms of complications.^4,23^ Our results showed an increased risk of bleeding in the type 2–3 PAD group compared to both type 1 PAD and no PAD. This is most likely due to the greater success or attempts to cannulate a papilla in type 2–3 PAD, but could also be explained by more frequent sphincterotomies. By contrast, with type 1 PAD, further cannulation attempts may be abandoned due to the inability to cannulate or fear of complications, which, in turn, may impede the risk of complications. This difference could also be explained by the difficulty in visualizing the papilla, which may be hidden from the endoscopist’s view within the diverticulum, and cannulation attempts might be abandoned. We also found no difference in procedure time between the groups, which could further explain the difficulty and abandoned cannulation attempts in type 1 PAD. With this theory, cannulation success and potential adverse events would be inversely related. This relationship may account for the different results regarding complications in previous studies: a type 1 PAD could theoretically pose a higher risk of adverse events if successful cannulation was achieved or attempted at all costs. Also, the risk of adverse events could theoretically be inversely related to experience with the novice abandoning a difficult procedure early without complications. However, beyond adjusting for precut and regular sphincterotomy, we were unable to determine the number and nature of cannulation attempts, or whether such attempts were aborted. Also, we have not been able to adjust for several other potential confounding factors possibly influencing the success of cannulation and adverse events such as the type of papillae and the experience of the endoscopist.

## Conclusion

Our study demonstrates that PAD occurs in approximately 10% of ERCP procedures and that cannulation success appears negatively affected by the presence of type 1 PAD. We found no differences in the rate of total adverse events, except for an increased risk of bleeding with type 2–3 PAD. However, the rate of adverse events with type 1 PAD is likely influenced by the inability to achieve successful cannulation, which thereby reduces the risk of complications.

## Supplemental Material

sj-pdf-1-tag-10.1177_17562848241279105 – Supplemental material for The impact of periampullary diverticula on cannulation and adverse events in endoscopic retrograde cholangiopancreatography Supplemental material, sj-pdf-1-tag-10.1177_17562848241279105 for The impact of periampullary diverticula on cannulation and adverse events in endoscopic retrograde cholangiopancreatography by Arvid Gustafsson, Bobby Tingstedt and Greger Olsson in Therapeutic Advances in Gastroenterology

sj-pdf-2-tag-10.1177_17562848241279105 – Supplemental material for The impact of periampullary diverticula on cannulation and adverse events in endoscopic retrograde cholangiopancreatography Supplemental material, sj-pdf-2-tag-10.1177_17562848241279105 for The impact of periampullary diverticula on cannulation and adverse events in endoscopic retrograde cholangiopancreatography by Arvid Gustafsson, Bobby Tingstedt and Greger Olsson in Therapeutic Advances in Gastroenterology

sj-pdf-3-tag-10.1177_17562848241279105 – Supplemental material for The impact of periampullary diverticula on cannulation and adverse events in endoscopic retrograde cholangiopancreatography Supplemental material, sj-pdf-3-tag-10.1177_17562848241279105 for The impact of periampullary diverticula on cannulation and adverse events in endoscopic retrograde cholangiopancreatography by Arvid Gustafsson, Bobby Tingstedt and Greger Olsson in Therapeutic Advances in Gastroenterology

## Declarations

**Ethics approval and consent to participate:** This study was approved by the Swedish Ethical Review Authority (DNR: 2021-06089-01).

**Consent for publication:** Not applicable.

**Availability of data and materials:** Not applicable.
