# Supplementary material for: The impact of periampullary diverticula on cannulation and adverse events in endoscopic retrograde cholangiopancreatography
Source: Ther Adv Gastroenterol. 2024 Oct 5;17:17562848241279105. doi: 10.1177/17562848241279105 (PMC11470493; doi:10.1177/17562848241279105)
Supplement: sj-pdf-1-tag-10.1177_17562848241279105 – Supplemental material for The impact of periampullary diverticula on cannulation and adverse events in endoscopic retrograde cholangiopancreatography [file sj-pdf-1-tag-10.1177_17562848241279105.pdf]

Variable	Bleeding Cases (%)	Bleeding OR (95% CI)	Cholangitis Cases (%)	Cholangitis OR (95% CI)
**PAD**				
Type 1	41 (1.6)	1.20 (0.87-1.64)<br>1.12 (0.81-1.54)	44 (1.7)	0.76 (0.56-1.02)<br>0.80 (0.59-1.08)
Type 2-3	108 (2)	1.52 (1.24-1.87)<br>1.41 (1.15-1.74)	104 (1.9)	0.86 (0.71-1.06)<br>0.90 (0.73-1.10)
No PAD	762 (1.3)	Ref	1,284 (2.2)	Ref
**sex**				
female	468 (1.3)	0.92 (0.81-1.05)<br>0.93 (0.81-1.06)	699 (2)	0.83 (0.74-0.92)<br>0.88 (0.79-0.98)
male	443 (1.4)	Ref	733 (2.4)	Ref
**age**				
>82	258 (1.6)	1.17 (0.97-1.39)	338 (2.1)	0.99 (0.85-1.15)
73-81	219 (1.3)	0.98 (0.82-1.18)	352 (2.1)	1.03 (0.88-1.19)
62-72	200 (1.3)	0.94 (0.78-1.14)	380 (2.4)	1.16 (1.01-1.35)
<=61	232 (1.4)	Ref	357 (2.1)	Ref
**ASA**				
3-4	385 (1.7)	1.38 (1.21-1.57)<br>1.34 (1.17-1.54)	533 (2.3)	1.11 (1.00-1.24)
1-2	526 (1.2)	Ref	899 (2.1)	Ref
**indication**				
stone	274 (1.1)	1.25 (1.07-1.47)<br>1.39 (1.18-1.64)	319 (1.3)	0.42 (0.37-0.48)<br>0.46 (0.40-0.52)
cholangitis	124 (2.1)	1.87 (1.51-2.32)<br>1.64 (1.31-2.05)	205 (3.4)	1.15 (0.98-1.35)<br>1.22 (1.04-1.43)
pancreatitis	24 (0.7)	0.66 (0.44-1.01)<br>0.69 (0.46-1.06)	42 (1.3)	0.43 (0.31-0.59)<br>0.48 (0.35-0.65)
J/M	274 (1.1)	Ref	732 (3)	Ref
**setting**				
acute	722 (1.5)	1.48 (1.26-1.74)<br>1.56 (1.32-1.85)	1,059 (2.2)	1.09 (0.97-1.23)
elective	189 (1)	Ref	373 (2)	Ref
**sphincterotomy**				
precut	147 (1.9)	1.40 (1.17-1.67)<br>1.35 (1.12-1.63)	166 (2.1)	1.02 (0.87-1.21)<br>0.81 (0.68-0.96)
no	90 (1)	0.76 (0.61-0.94)<br>0.74 (0.59-0.93)	229 (2.6)	1.27 (1.10-1.46)
yes	667 (1.4)	Ref	1,022 (2.1)	Ref
**procedure time**				
>48	266 (1.6)	1.43 (1.19-1.71)<br>1.29 (1.10-1.52)	474 (2.9)	1.95 (1.68-2.26)<br>1.78 (1.53-2.08)
31-47	232 (1.5)	1.30 (1.08-1.57)<br>1.20 (1.01-1.41)	366 (2.4)	1.57 (1.35-1.84)<br>1.46 (1.25-1.72)
21-30	194 (1.3)	1.10 (0.91-1.34)	304 (2)	1.32 (1.12-1.55)<br>1.25 (1.06-1.48)
<=20	219 (1.2)	Ref	288 (1.5)	Ref

Bleeding

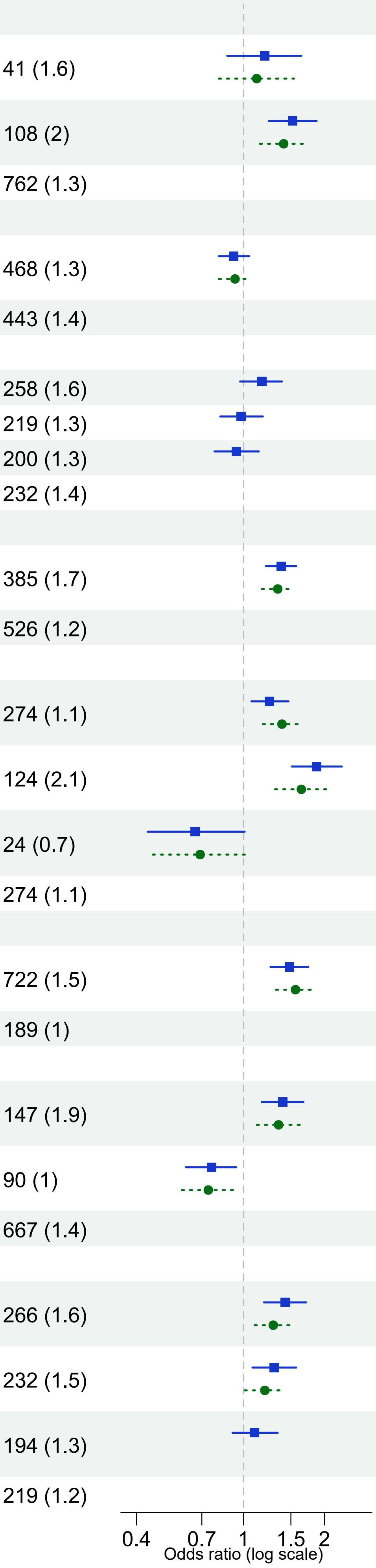

Cholangitis

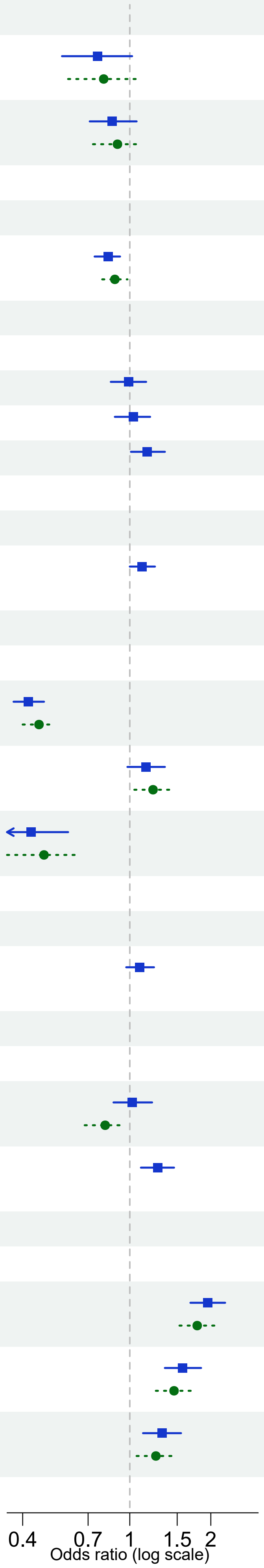

Univariable ● Multivariable

Model 1: χ2(df = 13, n = 65,041) = 162.258, P < .001, Nagelkerke R2 = 1.8%, classification = 98.6%

Model 2: χ2(df = 11, n = 65,041) = 300.329, P < .001, Nagelkerke R2 = 2.4%, classification = 97.8%
