# Supplementary material for: The impact of periampullary diverticula on cannulation and adverse events in endoscopic retrograde cholangiopancreatography
Source: Ther Adv Gastroenterol. 2024 Oct 5;17:17562848241279105. doi: 10.1177/17562848241279105 (PMC11470493; doi:10.1177/17562848241279105)
Supplement: sj-pdf-2-tag-10.1177_17562848241279105 – Supplemental material for The impact of periampullary diverticula on cannulation and adverse events in endoscopic retrograde cholangiopancreatography [file sj-pdf-2-tag-10.1177_17562848241279105.pdf]

Variable	Successful cannulation: Cases (%)	Successful cannulation: OR (95% CI)	Adverse events: Cases (%)	Adverse events: OR (95% CI)
PAD				
Type 1	2,114 (80.1)	0.52 (0.47-0.57)<br>0.42 (0.38-0.46)	381 (14.6)	0.86 (0.77-0.97)<br>0.98 (0.88-1.10)
Type 2-3	5,035 (91.7)	1.41 (1.28-1.56)<br>1.20 (1.08-1.32)	867 (16.0)	0.96 (0.89-1.04)<br>1.08 (1.00-1.17)
No PAD	52,091 (88.7)	Ref	9,570 (16.5)	Ref
sex				
female	31,795 (89.0)	1.09 (1.04-1.14)<br>1.03 (0.98-1.08)	5,874 (16.6)	1.04 (1.00-1.09)<br>1.05 (1.01-1.09)
male	27,433 (88.1)	Ref	4,941 (16.1)	Ref
age				
>82	14,787 (89.0)	0.83 (0.78-0.89)<br>0.86 (0.80-0.93)	2,075 (12.6)	0.52 (0.49-0.55)<br>0.60 (0.56-0.64)
73-81	14,558 (87.1)	0.70 (0.65-0.75)<br>0.76 (0.71-0.82)	2,401 (14.5)	0.61 (0.58-0.65)<br>0.69 (0.65-0.73)
62-72	13,931 (87.3)	0.70 (0.66-0.76)<br>0.80 (0.75-0.86)	2,581 (16.4)	0.71 (0.67-0.75)<br>0.77 (0.73-0.82)
<=61	15,819 (90.7)	Ref	3,736 (21.8)	Ref
ASA				
3-4	20,187 (86.7)	0.76 (0.73-0.80)<br>0.82 (0.78-0.86)	3,384 (14.7)	0.83 (0.79-0.86)<br>0.93 (0.89-0.98)
1-2	39,053 (89.5)	Ref	7,434 (17.3)	Ref
indication				
stone	23,621 (92.4)	2.39 (2.26-2.53)<br>2.38 (2.24-2.52)	3,753 (14.8)	1.00 (0.96-1.06)<br>1.08 (1.03-1.14)
cholangitis	5,742 (94.1)	3.15 (2.82-3.52)<br>3.41 (3.04-3.82)	890 (14.8)	1.00 (0.92-1.08)<br>1.09 (1.00-1.18)
pancreatitis	2,904 (88.2)	1.47 (1.31-1.64)<br>1.41 (1.26-1.57)	389 (12.0)	0.78 (0.70-0.88)<br>0.79 (0.71-0.89)
J/M	20,927 (83.6)	Ref	3,650 (14.8)	Ref
setting				
acute	42,753 (88.6)	1.00 (0.95-1.05)<br>1.09 (1.03-1.15)	7,965 (16.7)	1.09 (1.04-1.14)<br>1.37 (1.31-1.45)
elective	16,487 (88.6)	Ref	2,853 (15.6)	Ref
sphincterotomy				
precut			1,454 (18.8)	1.22 (1.15-1.30)<br>1.08 (1.01-1.15)
no			1,474 (17.0)	1.08 (1.02-1.15)<br>0.93 (0.87-0.99)
yes			7,751 (15.9)	Ref
procedure time				
>48			3,308 (20.4)	1.70 (1.61-1.80)<br>1.67 (1.57-1.77)
31-47			2,704 (17.5)	1.41 (1.33-1.49)<br>1.42 (1.34-1.51)
21-30			2,320 (15.2)	1.19 (1.12-1.27)<br>1.19 (1.12-1.27)
<=20			2,483 (13.1)	Ref

Successful cannulation

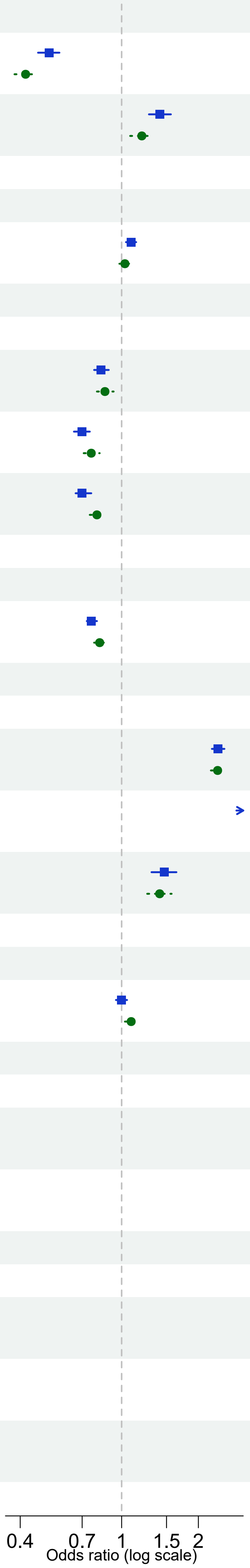

Adverse events

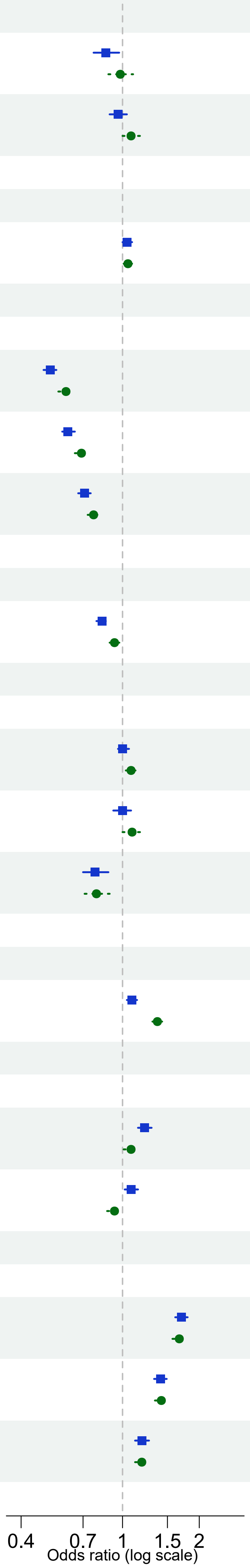

Univariable · Multivariable

Model 1: χ2(df = 11, n = 70,668) = 14909.40, P < .001, Nagelkerke R2 = 38.6%, classification = 89.6%

Model 2: χ2(df = 11, n = 71,531) = 1698.46, P < .001, Nagelkerke R2 = 4.0%, classification = 83.7%
