# Supplementary material for: The impact of periampullary diverticula on cannulation and adverse events in endoscopic retrograde cholangiopancreatography
Source: Ther Adv Gastroenterol. 2024 Oct 5;17:17562848241279105. doi: 10.1177/17562848241279105 (PMC11470493; doi:10.1177/17562848241279105)
Supplement: sj-pdf-3-tag-10.1177_17562848241279105 – Supplemental material for The impact of periampullary diverticula on cannulation and adverse events in endoscopic retrograde cholangiopancreatography [file sj-pdf-3-tag-10.1177_17562848241279105.pdf]

Variable	Pancreatitis Cases (%)	Pancreatitis OR (95% CI)	Perforation Cases (%)	Perforation OR (95% CI)
PAD				
Type 1	95 (3.6)	0.67 (0.55-0.83)<br>0.82 (0.66-1.01)	19 (0.7)	1.12 (0.70-1.78)<br>1.11 (0.70-1.77)
Type 2-3	257 (4.7)	0.89 (0.78-1.01)<br>1.03 (0.90-1.18)	44 (0.8)	1.25 (0.91-1.71)<br>1.29 (0.94-1.77)
No PAD	3,075 (5.3)	Ref	377 (0.6)	Ref
sex				
female	2,012 (5.7)	1.25 (1.17-1.34)<br>1.25 (1.16-1.34)	250 (0.7)	1.15 (0.95-1.39)<br>1.17 (0.97-1.42)
male	1,414 (4.6)	Ref	190 (0.6)	Ref
age				
>82	533 (3.2)	0.39 (0.35-0.43)<br>0.45 (0.41-0.51)	85 (0.5)	0.57 (0.44-0.75)
73-81	721 (4.4)	0.53 (0.48-0.58)<br>0.58 (0.53-0.64)	102 (0.6)	0.69 (0.53-0.88)
62-72	798 (5.1)	0.62 (0.56-0.67)<br>0.66 (0.60-0.72)	99 (0.6)	0.70 (0.54-0.90)
<=61	1,370 (8)	Ref	154 (0.9)	Ref
ASA				
3-4	919 (4)	0.67 (0.62-0.73)<br>0.84 (0.77-0.91)	141 (0.6)	0.88 (0.72-1.08)
1-2	2,508 (5.8)	Ref	299 (0.7)	Ref
indication				
stone	1,342 (5.3)	1.12 (1.04-1.22)<br>1.13 (1.04-1.22)	161 (0.6)	1.22 (0.97-1.54)<br>1.46 (1.17-1.82)
cholangitis	199 (3.3)	0.68 (0.59-0.80)	27 (0.4)	0.86 (0.57-1.30)
pancreatitis	143 (4.4)	0.92 (0.77-1.10)	15 (0.5)	0.88 (0.52-1.51)
J/M	1,173 (4.7)	Ref	129 (0.5)	Ref
setting				
acute	2,154 (4.5)	0.63 (0.59-0.68)<br>0.75 (0.70-0.81)	285 (0.6)	0.71 (0.58-0.86)
elective	1,273 (6.9)	Ref	155 (0.8)	Ref
sphincterotomy				
precut	534 (6.9)	1.41 (1.28-1.55)	75 (1)	1.66 (1.29-2.15)<br>1.31 (1.01-1.70)
no	432 (5)	1.00 (0.90-1.11)	75 (0.9)	1.48 (1.15-1.91)<br>1.27 (0.98-1.65)
yes	2,432 (5)	Ref	285 (0.6)	Ref
procedure time				
>48	1,223 (7.5)	2.29 (2.08-2.53)<br>2.30 (2.08-2.55)	190 (1.2)	3.61 (2.71-4.82)<br>3.42 (2.55-4.61)
31-47	871 (5.6)	1.68 (1.51-1.86)<br>1.73 (1.56-1.93)	112 (0.7)	2.22 (1.63-3.04)<br>2.17 (1.58-2.97)
21-30	681 (4.5)	1.31 (1.18-1.47)<br>1.34 (1.20-1.49)	76 (0.5)	1.53 (1.09-2.14)<br>1.51 (1.07-2.12)
<=20	652 (3.4)	Ref	62 (0.3)	Ref

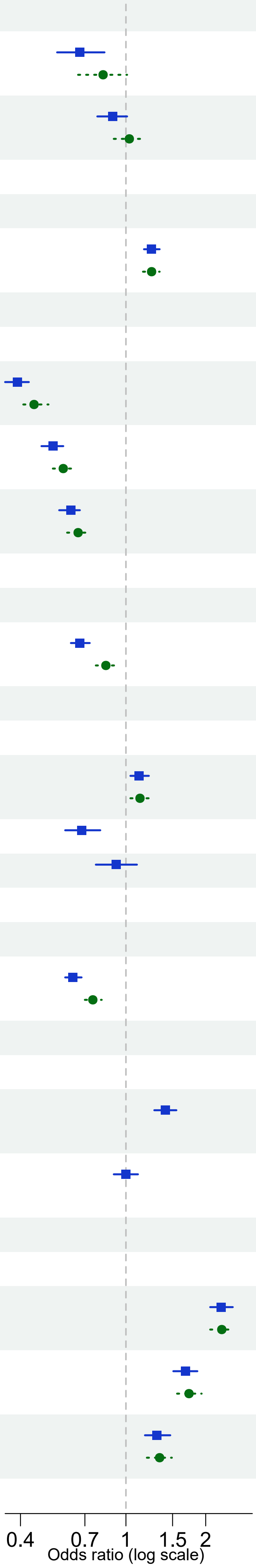

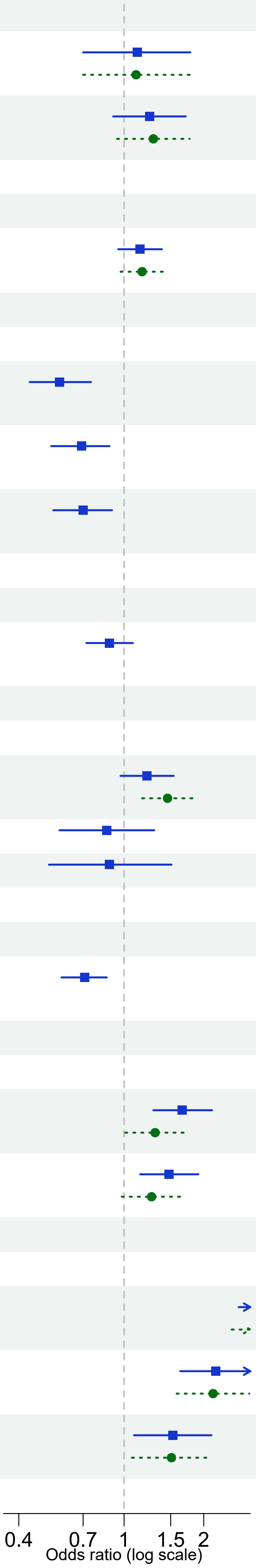

Model 1: χ2(df = 15, n = 65,041) = 936.390, P < .001,
Nagelkerke R2 = 4.0%, classification = 94.8%
Model 2: χ2(df = 10, n = 65,041) = 181.993, P < .001,
Nagelkerke R2 = 4.0%, classification = 99.3%

Univariable Multivariable
